# Novel gas exposure system for the controlled exposure of plants to gaseous hydrogen fluoride

**DOI:** 10.1007/s10661-023-11382-8

**Published:** 2023-05-29

**Authors:** Katherine F. DeMille, Stephen D. Emsbo-Mattingly, Gary Krieger, Michael Howard, Katie B. Webster, Michelle DaCosta

**Affiliations:** 1 NewFields Environmental Forensics Practice, LLC, 300 Ledgewood Place, Suite 205, Rockland, MA 02370 USA; 2 NewFields E&E, Longmont, CO 80503 USA; 3 Mission Support and Test Services, LLC, Las Vegas, NV 89193 USA; 4 University of Massachusetts Amherst, 310 Paige Laboratory, 161 Holdsworth Way, Amherst, MA 01003 USA

**Keywords:** Bioindicator, Gas exposure system, Hydrogen fluoride, Air monitoring

## Abstract

Plants can serve as sensitive bioindicators of the presence of contaminant vapors in the atmosphere. This work describes a novel laboratory-based gas exposure system capable of calibrating plants as bioindicators for the detection and delineation of the atmospheric contaminant hydrogen fluoride (HF) as a preparatory step for monitoring release emissions. To evaluate changes in plant phenotype and stress-induced physiological effects attributed to HF alone, the gas exposure chamber must have additional controls to simulate otherwise optimal plant growth conditions including variables such as light intensity, photoperiod, temperature, and irrigation. The exposure system was designed to maintain constant growth conditions during a series of independent experiments that varied between optimal (control) and stressful (HF exposure) conditions. The system was also designed to ensure the safe handling and application of HF. An initial system calibration introduced HF gas into the exposure chamber and monitored HF concentrations by cavity ring-down spectroscopy for a 48-h period. Stable concentrations inside the exposure chamber were observed after approximately 15 h, and losses of HF to the system ranged from 88 to 91%. A model plant species (*Festuca arundinacea*) was then exposed to HF for 48 h. Visual phenotype stress-induced responses aligned with symptoms reported in the literature for fluoride exposure (tip dieback and discoloration along the dieback transition margin). Fluoride concentrations in exposed tissues compared to control tissues confirmed enhanced fluoride uptake due to HF exposure. The system described herein can be applied to other reactive atmospheric pollutants of interest in support of bioindicator research.

## Introduction

Natural and anthropogenic sources, such as volcanic emissions, coal-fired power plants, aluminum smelters, phosphate fertilizer plants, refineries, plastic factories, and nuclear fuel process facilities, can potentially emit HF into the atmosphere (ATSDR, 2003; Cheng, 2018). Hydrogen fluoride releases pose risk to both human health and environmental receptors. Acute exposure to gaseous HF can result in severe respiratory damage, eye irritation, and dermal burns (USEPA, 2016; ATSDR, 2003). In terms of environmental impacts, fluoride is reported to be the most phytotoxic atmospheric pollutant (Weinstein & Davison, 2003). The detection of accidental or ephemeral industrial HF releases is challenging due to the reactivity of HF and the episodic nature of such releases that may elude periodic air monitoring. Understanding exposure symptoms in vegetation native to areas of release provides an alternative approach for evaluating the footprint of HF impact.

Plants serve as sentinel species for detecting contaminants in the air. Researchers have demonstrated that plants exhibit signs of stress when exposed to certain contaminant vapors, such as HF, at concentrations well below levels considered harmful to human receptors. For example, HF-induced physiological impacts on plants have been observed at concentrations below 1 ppbv, well below the US Occupational Safety and Health Administration (OSHA) permissible exposure limit for HF in the air (3 ppmv) (Weinstein & Davidson, 2004; Weinstein et al., 1990). Prior studies, cited below, have demonstrated HF-induced stress at low atmospheric concentrations; however, limited information is provided on threshold concentrations and biochemical pathways for a broad range of plants that are well-suited for use as bioindicators in temperate ecosystems.

Symptoms of plant fluoride injury have been documented since the mid-nineteenth century. HF is reported to be 10 to 100 times more toxic to vegetation than other common atmospheric pollutants (Weinstein & Davidson, 2004). Observed changes in plant phenotype due to fluoride stress include tip dieback, marginal and interveinal chlorosis, and anthocyanosis (Weinstein and Davidson, 2003; Weinstein & Davidson, 2004). Plants that display measurable, reproducible symptoms of HF exposure that can be distinguished from other environmental stressors can serve as candidate bioindicators for HF.

Recent studies on the use of plants as bioindicators for fluoride emissions are largely focused on placing greenhouse-cultivated plants proximal to known sources of fluoride emissions (Fornasiero, 2003; Divan Jr. et al., 2007; Rey-Asensio & Carballeira, 2007; Divan Jr. et al., 2008; Rodriguez et al., 2015; Franzaring et al., 2007; Louback et al., 2016; Sant’Anna-Santos et al., 2021). These studies establish that bioindicators can provide consistent changes in phenotype in response to HF exposure. The studies also establish that there is a dose–response relationship between visual symptoms and fluoride dose. However, responses are species-specific and can be confounded by other abiotic stressors, such as water and nutrient deficiencies, and biotic stressors, such as insect herbivores or pathogens (Weinstein & Davison, 2003). Further research is needed to establish species-specific dose–response and delineate HF-induced stress response from other common environmental stressors.

Controlled laboratory-based research allows for the isolation and sequential introduction of variables to better understand HF-induced plant response. Published laboratory studies of gaseous HF exposures date back to the 1960s and 1970s and involve various types of fumigation chambers to demonstrate HF effects on different plants such as various bean species (*Phaseolus vulgaris, P. lunatus*), maize (*Zea mays*), citrus (*Citrus sinensis*, *C. paradisi,* and *C. unshiu*), tobacco (*Nicotiana tabacum*), rice (*Oryza sativa*), and lichens (*Cladonia cristatella*, *C. polycarpoides*, and *Parmelia plittii*) (Adams, 1961; Adams et al., 1957; Döğeroğlu et al., 2003; MacLean et al., 1968; Matsushima & Brewer, 1972; McCune et al., 1964; Nash, 1971; Pack, 1971; Sun & Su, 1985). Exposure periods varied from hours to weeks, with HF concentrations ranging from 1 ppbv to 10 ppmv. Many of these studies were limited at the time by available analytical methodologies and instrumentation for measuring gaseous HF. For example, HF exposure concentrations were often quantified by titration or by limed filter paper, a method intended to estimate the rate of atmospheric fluoride deposition (Adams, 1957; Pack, 1971; Smith, 1988). Moreover, the fumigation chambers in earlier studies were not optimized for controlling plant growth conditions such as light, temperature, and humidity throughout HF exposure, resulting in limitations in extrapolating mechanisms of HF uptake and HF-induced stress effects for potential bioindicator plants in field environments.

This work features a custom-built gas exposure system designed for laboratory-based HF exposure studies. A plant growth chamber capable of light, temperature, and humidity control was converted into a gas exposure system. The exposure system was equipped with two independent analyzers that provided HF monitoring in the range of 20 pptv to 12 ppmv. The system was designed to facilitate the safe handling and application of gaseous HF. Two 48-h calibration experiments were conducted to quantify wall losses to the system. A model grass moderately tolerant to HF (tall fescue, *Festuca arundinacea*) and two tropical plants previously reported to be highly sensitive to HF were then placed inside the exposure chamber and dosed with HF for a 48-h period to assess system performance.

## Description of the gas exposure system

### Modified growth chamber

To evaluate plant response(s) to HF impact alone, it is critical that test plants are not inadvertently introduced to additional stressors that may arise from sub-optimal growth conditions. Plant growth chambers are specifically designed to optimize plant growth and provide control over variables such as light intensity, photoperiod, temperature, and relative humidity. A Percival model LED-36HVL growth chamber was modified in collaboration with Percival applications engineers with the intent to convert the growth chamber into a gas exposure system (Percival Scientific, Perry, Iowa). The growth chamber was approximately 2 m in height, 0.9 m in depth, and 1 m in width, providing 0.8 m^3^ of interior space. The lighting system included cool white, red, far-red, and blue LEDs that were independently dimmable between 10 and 100% to achieve a target photosynthetically active radiation (measured as photosynthetic photon flux density, PPFD). Lamps were located on chamber side panels, separated from the chamber interior by glass panels, thereby reducing internal heat. The interior temperature was controlled by an air-cooled condensing unit and a ceiling-mounted circulation fan. When lights were on to simulate a daytime cycle, temperature settings between 5 °C and 44 °C + / − 0.5 °C were achievable. Relative humidity was controlled indirectly through the irrigation system and measured by an electronic relative humidity sensor. Growth chamber data such as setpoints and process values were automatically stored at one-minute intervals using Intellus Ultra Connect software (Percival Scientific, Perry, Iowa).

**Fig. 1:**
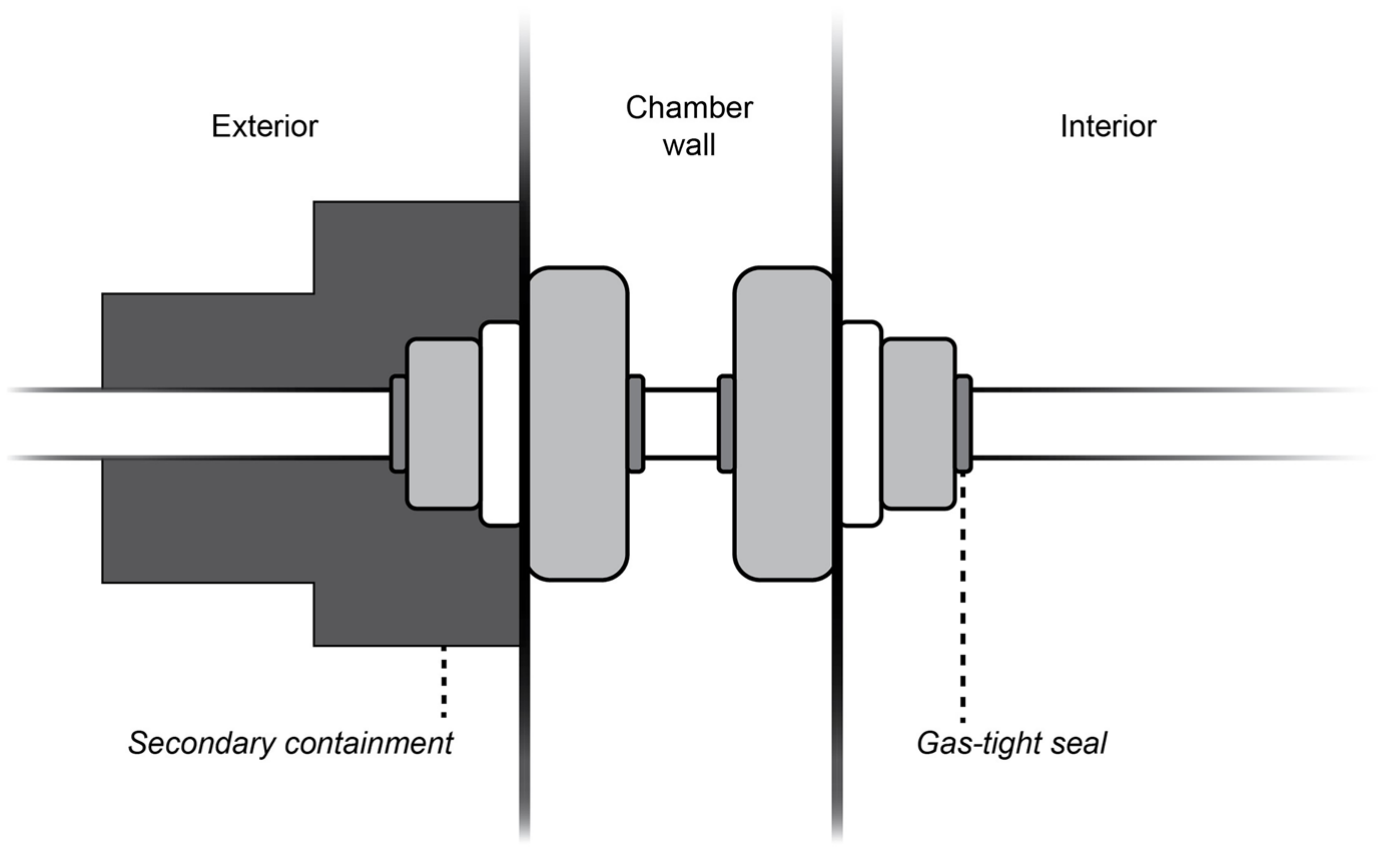
Diagram of gas entry and exit ports. PFA tubing passed through the growth chamber sidewall through two Swagelok PFA fittings that comprised each port. A gas-tight seal was achieved on the 0.95 cm side of the reducing union exposed on the chamber interior

The Percival growth chamber was customized to include a gas entry port and a gas exit port. Ports were placed above the left side panel light fixture. Each port was comprised of two Swagelok perfluoroalkoxy (PFA) 1.27 cm to 0.95 cm reducing unions modified to allow for the pass-through of 0.95 cm outer diameter (OD) Swagelok PFA tubing. Tubing was fed through the chamber sidewall, passing through both Swagelok-reducing unions. A gas-tight seal was achieved on the 0.95 cm side of the reducing union exposed on the interior chamber wall (Fig. 1).

Moderate HF-induced corrosion inside the growth chamber was anticipated during the system design phase. To mitigate this issue, the interior refrigeration coils were coated in a phenolic coating to prevent acid corrosion. However, despite system modifications, significant losses to the system were observed during early operations due to the highly reactive character of HF (i.e., HF was not detected in gas streams exiting the growth chamber). The supply and return gas lines were connected to bypass the growth chamber. HF concentration in the return gas stream was measured and compared to the HF concentration in the supply gas. A negligible difference between the two measurements was observed (< 5 ppbv). This confirmed that HF losses occurred inside the growth chamber and not the PFA tubing. The interior of the growth chamber contained glass paneling and metal components associated with the temperature control system. Glass and many types of metals react with HF; therefore, surfaces inside the growth chamber were not optimal for conducting HF exposure experiments.

**Fig. 2:**
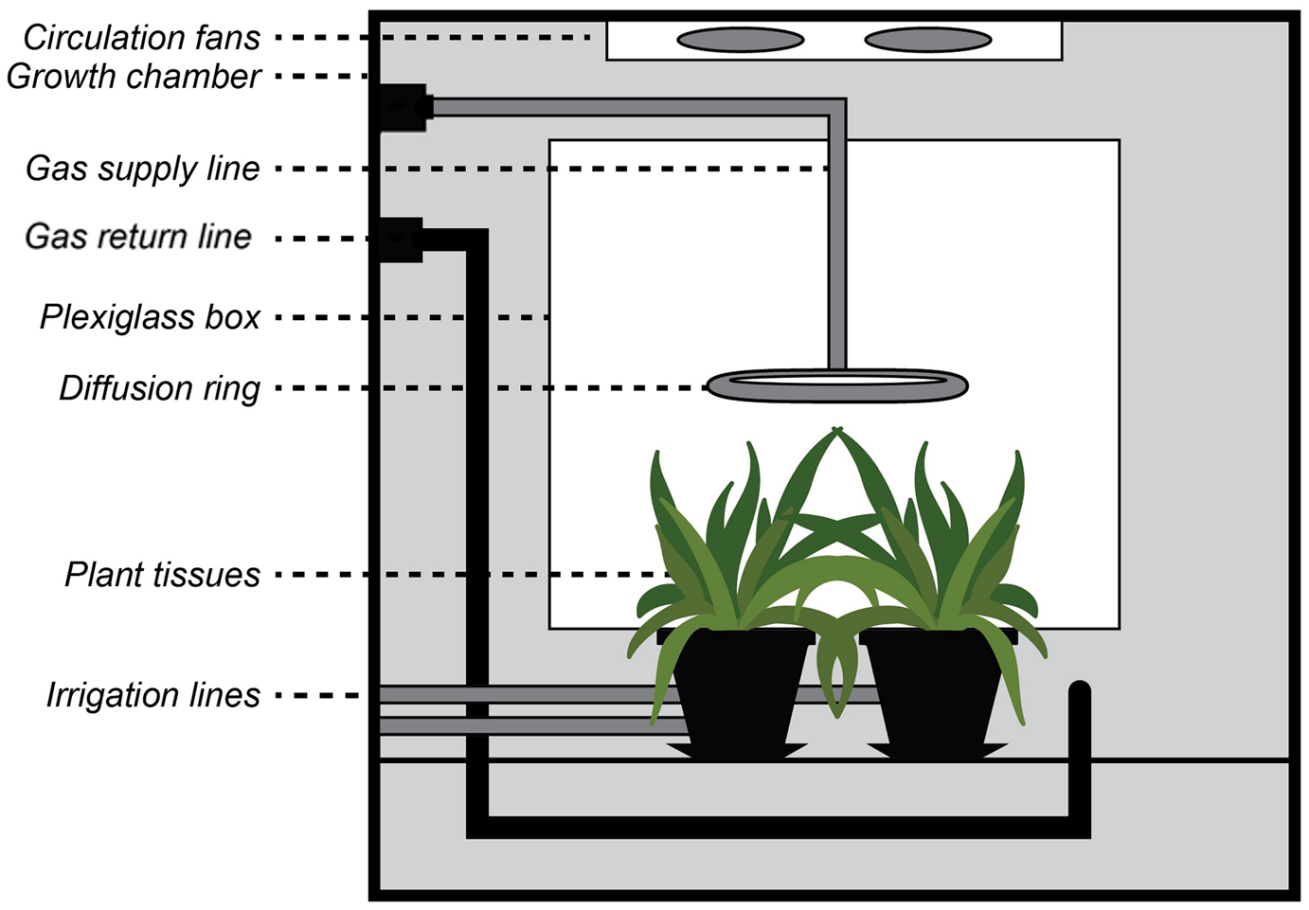
Diagram of the PEC

A plexiglass (polymethyl methacrylate) box was constructed to reduce losses inside the growth chamber. Plexiglass was selected over other materials because of its ability to transmit light and reported chemical compatibility with hydrofluoric acid at diluted concentrations (< 20% at 20 °C) (Industrial Specialties Mfg., 2022). Lights inside the growth chamber were turned on, and a LI-250A light sensor (LI-COR Biosciences, Lincoln, Nebraska) was used to measure PPFD which confirmed that sufficient light levels (450–550 µmol/m^2^/s) were achieved through the plexiglass for plant growth. A sealed 5-sided plexiglass box measuring 45.7 cm high, 45.7 cm wide, and 61.0 cm deep was then constructed. The removable bottom panel was outfitted with 12.7 cm diameter holes that allowed plant tissue inside the box, while the pot, soil, and irrigation tubes remained outside of the plexiglass box (Fig. 2). Gases were introduced into the plexiglass exposure chamber (PEC) through a 27.9 cm diameter diffusion ring comprised of 0.95 cm OD PFA tubing placed at a height just above plant canopies. As pressure increased inside the PEC, the gas escaped through holes surrounding the plant canopy. This design served to maximize the interaction between the supply gas and test plants. During HF exposure experiments, a vacuum line was placed outside of and below the PEC to keep the growth chamber at a slight negative pressure (see “Safety features” section below). The PEC was conditioned with 20 ppmv HF gas before the initiation of calibration experiments.

### Gas supply and return manifolds

Compressed gas cylinders of HF gas with nitrogen as the balance gas were formulated by SpecGas to a concentration of 5 ppmv HF and a concentration of 20 ppmv HF (SpecGas, Warminster, PA). Compressed HF cylinders (size 33A) were housed and secured inside the fumigation hood. Larger-sized ultrahigh purity (UHP) air cylinders (size 300, Airgas) were housed and secured next to the fumigation hood. HF was diluted with UHP air using the gas supply manifold to achieve target gas exposure concentrations. Flow rates for HF and air were modulated by mass flow controllers (Aalborg, Orangeburg, New York). Flow controllers were integrated into an automated control system with a custom operator interface (OPTO22, Temecula, California) to allow for the entry of set point flow rates and automated data storage.

**Fig. 3:**
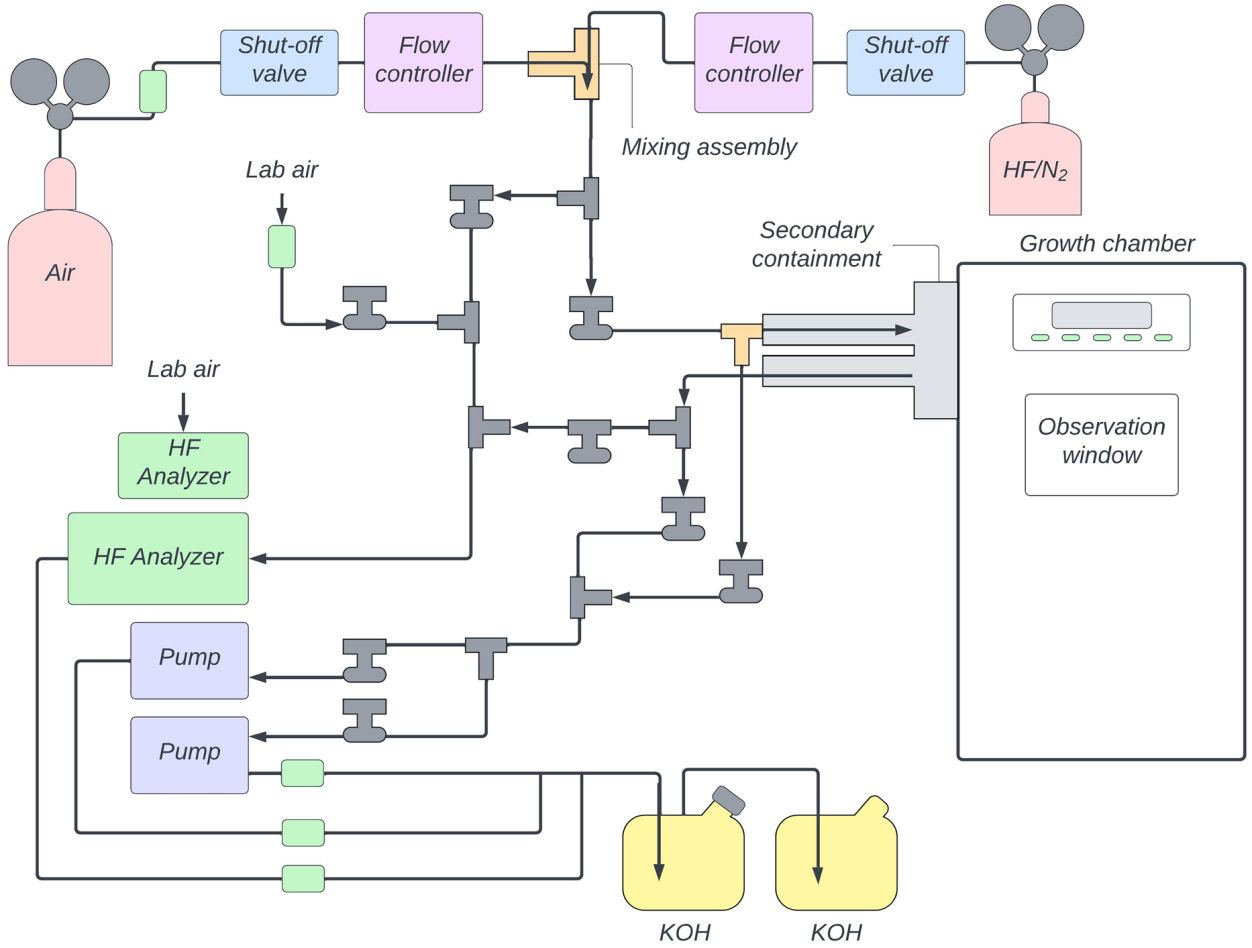
Diagram of the gas exposure system

Downstream of the mass flow controllers, HF and air flows were directed to a mixing assembly before the mixed gas entered the growth chamber (Fig. 3). The mixing assembly, constructed of stainless-steel Swagelok fittings, introduced the smaller flow of HF (up to 200 mL/min) downstream and in parallel to the larger flow of air (up to 2 L/min) to facilitate mixing. The system operators directed the gas mixture through the supply manifold to the HF detector for confirmation of supply gas concentrations.

Vacuum pumps removed gases continuously from the system during experiments. A smaller capacity vacuum pump was used during HF exposure periods (KNF N86 KTP Corrosion-Resistant Vacuum Pump, max flow rate of 5.5 standard liter per minute (slm)). A higher capacity secondary pump was applied at the end of exposure experiments to evacuate the system of HF at a faster rate (Welch PTE-Coated Vacuum Pump, max flow rate of 33.6 slm). Gases exiting the growth chamber were directed to the return gas manifold, which directed the return gas to either an HF detector or to caustic scrubber baths for neutralization. The scrubber baths consisted of two 9.5-L HDPE containers in series filled with 1 M KOH solution (Sigma Aldrich). A colorimetric, pH-sensitive indicator (phenolphthalein, Sigma Aldrich) was added to the buffer solutions to provide a visual means to monitor the remaining buffer capacity of the scrubber solutions.

### HF detection

The gas exposure system was outfitted with two independent HF detectors. A Tiger Optics T-I Max trace analyzer continuously measured HF concentrations by cavity ring-down spectroscopy within a range of 20 pptv to 1 ppmv HF (Tiger Optics, Horsham, Pennsylvania). A Honeywell Midas gas detector coupled with an HF sensor cartridge (Honeywell MIDAS-S-HFX) continuously measured HF concentrations by patented Chemcasette technology within a range of 1 and 12 ppmv (Honeywell, Charlotte, North Carolina). The detector configuration was adapted to measure HF concentrations in the supply gas, return gas, ambient HF levels inside the PEC, and ambient concentrations inside the laboratory for safety monitoring.

### Safety features

The gas exposure system included numerous safety features. During exposure experiments, a vacuum pump coupled with a needle valve removed gas from the growth chamber at a set flow rate. For safety purposes, the vacuum flow rate was set in slight excess of the total supply gas flow rate so that if a leak occurred, ambient laboratory air would be pulled inside the growth chamber. Automatic shut-off valves (Galtek normally closed solenoid valves) were placed in the supply lines between gas regulators and mass flow controllers. In the event of power failure, the automatic shutoff valves terminated the gas supply and prevented positive pressurization of the growth chamber. A magnehelic sensor was installed inside the growth chamber to monitor pressure throughout gas exposure experiments. Check valves were placed in gas lines to prevent reverse flow.

The supply and return gas manifolds were configured inside fumigation hoods. All PFA tubing that conveyed HF outside of the fumigation hoods was encased in stainless steel secondary containment. The secondary containment system also encased the supply and return gas ports. Secondary containment components were orbital welded for seamless connections. Annular space was continuously evacuated by a vacuum pump that directed gas to the caustic scrubber baths.

The gas exposure system was inspected for leaks before the initiation of HF use. A cylinder of compressed helium gas (UHP helium, Airgas) was connected to the supply lines. Helium was directed throughout the gas exposure system, and each fitting was verified to be gas-tight using a dielectric helium detector (SPX Dielectric Helium/Hydrogen MGD-2002 detector). The growth chamber was also monitored for leaks. Growth chamber seams that were not gas-tight were sealed with an industrial-grade sealant resistant to acid (Belzona Inc., Miami, Florida) before the introduction of HF.

Five surveillance cameras inside the laboratory permitted remote monitoring of gas regulators, the caustic scrubber baths, the laptop, and vacuum pumps. A laptop continuously recorded system measurements and provided remote access through AnyDesk software. Prior to entry into the laboratory, staff used this system to confirm that the HF concentration of the laboratory air was below 3 ppmv, the OSHA permissible exposure limit for HF in air. HF concentrations, measured by either the TI-Max or the Honeywell Midas analyzer, never exceeded ambient air levels of 0.1 ppbv to 1 ppmv (the detection limit of the Midas analyzer).

A supplementary alert system for power outages was procured for the laboratory independent of and in addition to the University’s notification system. An isocket (iSocket Systems, Varkaus, Finland) was powered by the same electrical circuit as exposure system equipment. If the isocket detected a power failure to the exposure chamber, it issued text message alerts to lab members as well as health and safety personnel.

## System calibration

Previous investigators have documented losses of reactive vapor phase compounds by mechanisms such as adsorption or chemical reactions that occurred on the walls of experimental chamber systems (Finlayson-Pitts & Pitts, 2000; Grosjean, 1985). Wall losses were expected in the gas exposure system due to the high surface-to-volume ratio of the PEC and moderate chemical compatibility reported between plexiglass and HF. A series of calibration experiments were conducted to quantify wall losses inside the PEC. Gas from the HF/N_2_ cylinder was first directed to the Honeywell Midas HF analyzer to measure concentration. HF/N_2_ and air were then introduced into the system at a flow rate of 100 mL/min 3.85 ppmv HF/N_2_ and 900 mL/min Ultra Zero Grade air, for a mixed supply concentration of 385 ppbv HF. Mixed gas was continuously supplied for a minimum of 48 h, which was previously demonstrated to induce visual stress symptoms on tall fescue leaves such as leaf tip die-back and chlorosis. After 48 h, the HF flow was discontinued. Airflow continued for an additional 24 h to facilitate purging the system of HF. Throughout the duration of the experiments, HF concentrations inside the plexiglass box were continuously monitored using the Tiger Optics TI-Max HF analyzer from a sampling point approximately 10 cm below the supply gas diffusion ring.

**Fig. 4:**
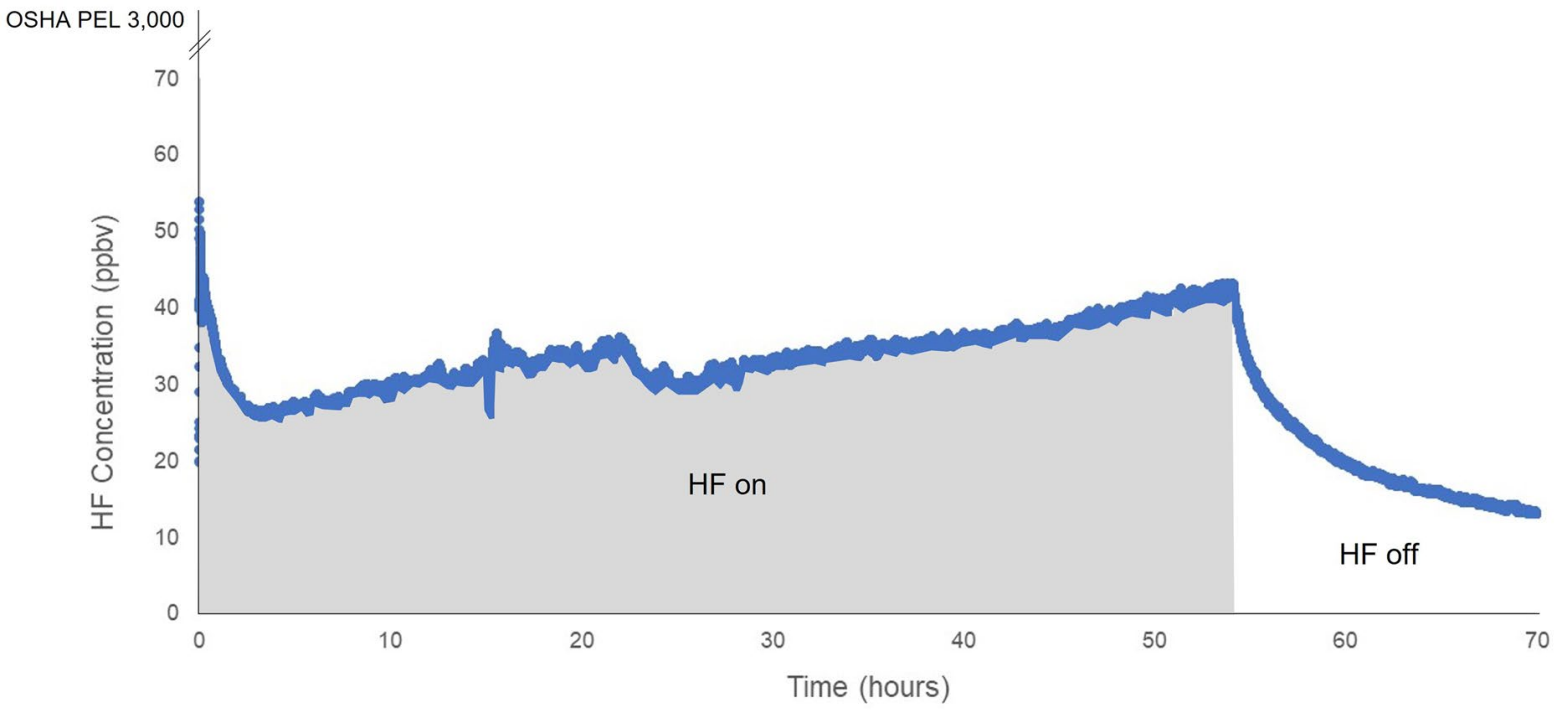
HF concentrations measured over time for calibration experiment 1

**Fig. 5:**
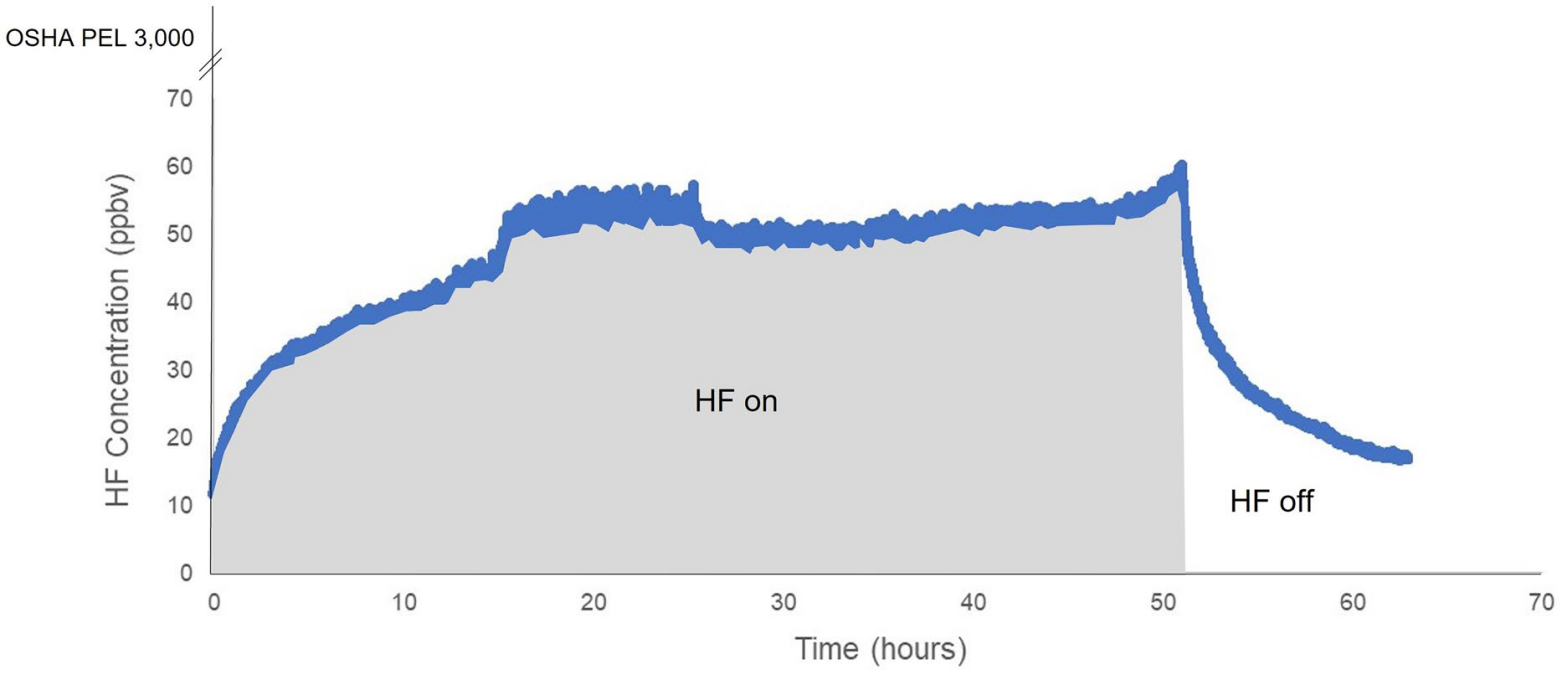
HF concentrations measured over time for calibration experiment 2

As depicted in Figs. 4 and 5, HF concentrations inside the PEC stabilized after approximately 15 h. Before the initiation of experiment 1, the exposure system had been used to measure the supply gas HF concentration. It appears that a slug of HF gas passed through the system as valves were opened during the start of the experiment. During this time, HF concentrations reached 53.7 ppbv before decreasing. The average HF reading was 33.4 + / − 4.12 ppbv while HF was flowing into the PEC. Generally, throughout experiment 1, the HF concentration exhibited a positive non-linear plateau distribution during the 48-h exposure period (Fig. 4).

The baseline reading for the HF analyzer was 0.2 ppbv HF. A baseline reading is a concentration that the analyzer reads before the introduction of HF into the system. Baseline measurements above zero were a result of coupling the highly sensitive TI-Max HF analyzer to the supply and exhaust gas manifolds comprised of fluorine-containing PFA tubing and fittings. Baseline measurements between 0.2 and 0.5 ppbv were observed prior to the introduction of any HF gas to the experimental system. Thus, it appears that low-level outgassing from PFA materials was the likely source of baseline readings, not the desorption of HF introduced into the gas exposure system. When the TI-Max analyzer was disconnected from the gas manifolds, HF measurements declined to approximately 0.005 ppbv, which was considered the ambient HF concentration in the laboratory air. Initial HF measurements at the start of experiments 1 and 2 were above baseline because the system was not completely purged of HF at the start of the experiments (Figs. 4 and 5).

The average HF concentration inside the PEC during calibration experiment 1 was 33.2 ppbv when corrected for baseline. Thus, in calibration experiment 1, an estimated 91% of HF in the supply gas was lost to the PEC. In the second calibration experiment, the average HF reading was 47.0 + / − 8.6 ppbv (Fig. 5). The average HF concentration inside the PEC during experiment 2 was 46.8 ppbv when corrected for baseline resulting in an estimated 88% loss of HF to the system.

Regardless of wall losses, HF concentrations inside PEC during calibration experiment 1 and experiment 2, at 33.2 ppbv and 46.8 ppbv, respectively, were sufficient for conducting future bioindicator research. Measurable fluoride uptake and physiological impacts have been evidenced at concentrations below 1 ppbv HF (Weinstein & Davidson, 2004; Weinstein et al., 1990). These calibration studies were used to correct the difference between the supply and exposure gas concentrations, as described below.

## Plant exposure experiment

Tall fescue was selected as the candidate bioindicator for research. Tall fescue is a common temperate perennial grass found in pastures, roadsides, and cultivated turf in many areas including North and South America, Europe, Asia, and Africa (Meyer & Watkins, 2003). In a previous study, tall fescue was demonstrated to accumulate high fluoride concentration in leaf tissue near a known fluoride emissions source without significant injury (Taylor et al., 1981). Therefore, tall fescue is a strong candidate bioindicator for HF. Tall fescue was seeded directly into a peat-based growing medium in 15.2 cm diameter and 15.2 cm depth pots. The seeded pots were maintained in the University of Massachusetts Research and Education Greenhouse (Amherst, MA) under optimal temperature conditions of 18–20 °C, watered daily, and fertilized weekly. After 6 weeks of establishment, plants were transferred to the PEC. Plants were adapted to PEC conditions for 3 days prior to the start of the experiment. Leaf tissues from three control tall fescue (pre-HF exposure) plants were harvested, dried in an oven at 65 °C, and analyzed for fluoride content at the University of Idaho Analytical Science Laboratory (Moscow, ID) following AOAC Method 975.04.

For the plant exposure experiment, the entire leaf canopy of one tall fescue plant was placed inside the PEC. Two additional plants previously reported to be highly sensitive to HF were also placed inside the PEC as in situ indicators of HF exposure (spider plant, *Chlorophytum comosum*, and corn plant, *Dracaena fragrans*) (Shahab et al., 2017). A flow rate of 1 L/min of 20 ppmv HF was directed into the PEC for 48 h. Based on the results of calibration experiments 1 and 2, HF concentrations inside the PEC were estimated to range from 1.8 to 2.4 ppmv. Throughout the exposure experiment, growth variables in the PEC remained at an average temperature of 25 + / − 0.04 °C, a light intensity of 500 µmol/m^2^/s, and an average percent relative humidity of 72 + / − 1%.

**Fig. 6:**
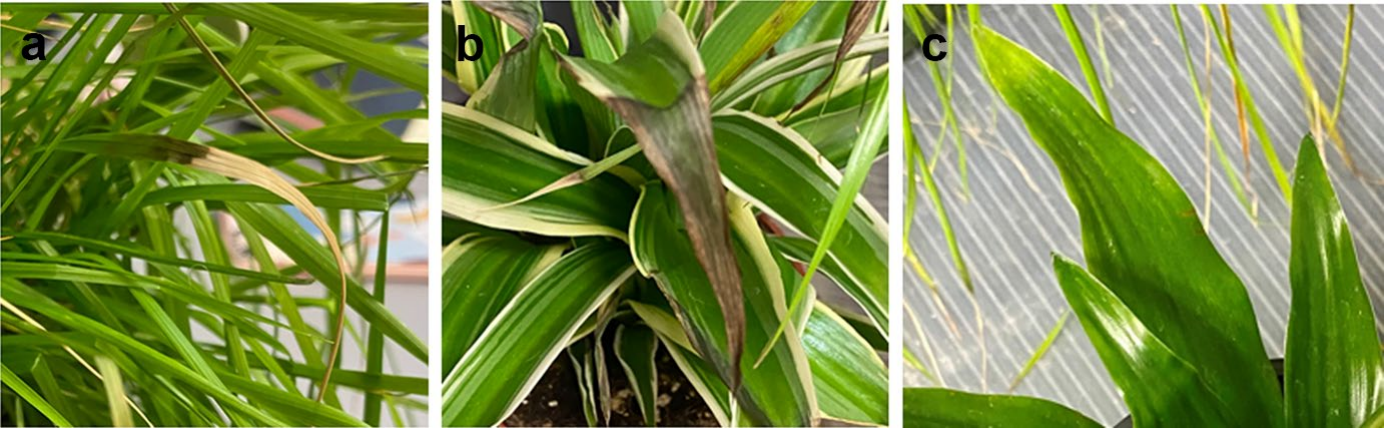
Images of tall fescue (**a**), spider plant (**b**), and corn plant (**c**) following a 48-h HF exposure

Within less than 24 h of HF exposure, visible symptoms of stress were observed, including leaf tip dieback, particularly in tall fescue leaf blades proximal to the HF source. Stress symptoms were also evident on the spider plant, which consisted of leaf tips turning black in color. Following 48-h exposure, a significant level of damage was observed in tall fescue leaves, with tip dieback extending to a majority of leaves of the canopy. Tip dieback and discoloration were observed in the spider plant, and some minor discoloration was observed in the leaf tips of the corn plant (Fig. 6).

**Fig. 7:**
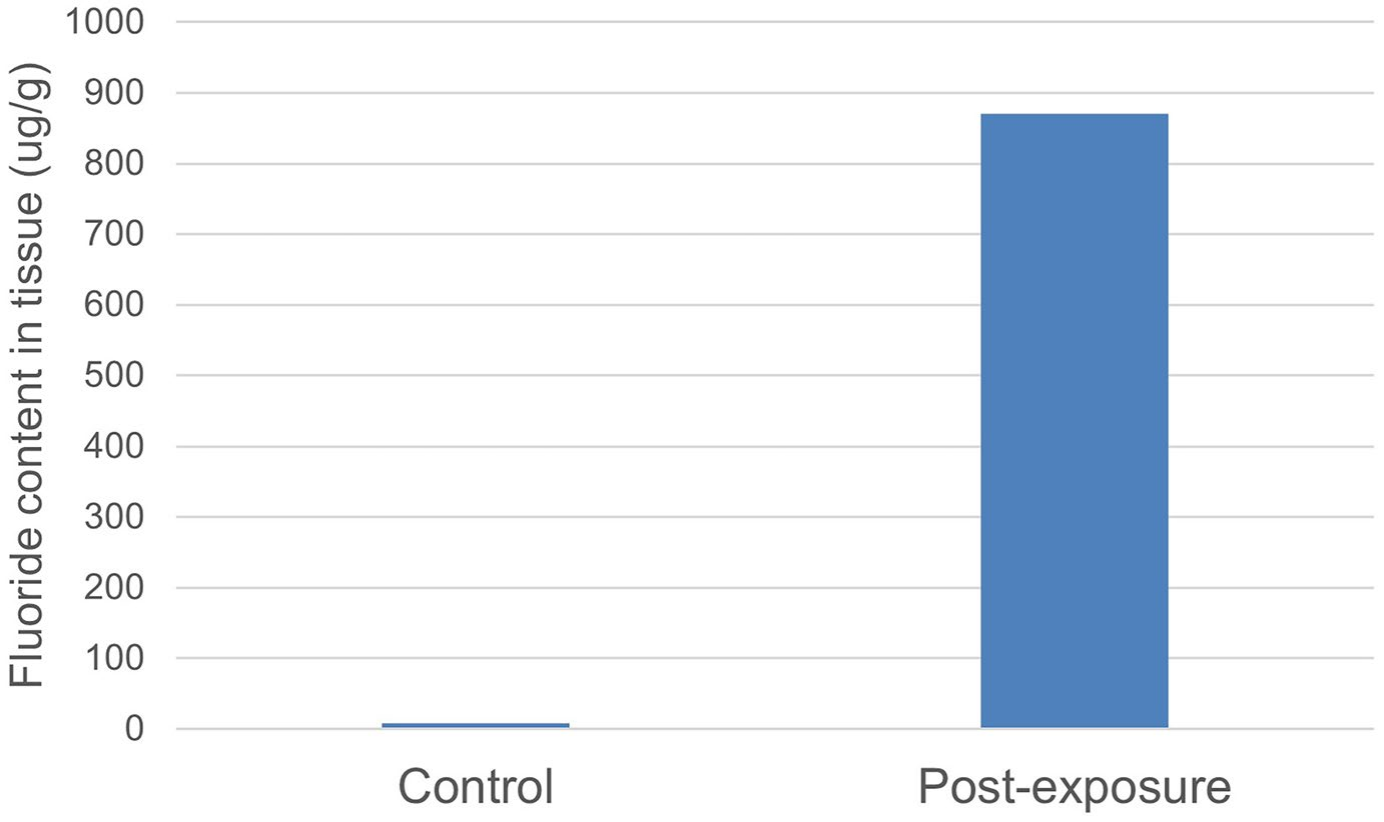
Tissue harvested from three control tall fescue plants did not contain measurable fluoride (< 8.5 µg/g). One tall fescue plant exposed to an HF/N_2_ mixture for 48 h had a tissue fluoride content of 870 µg F/g

At the end of the exposure experiment, tall fescue leaves were harvested and surface-washed to remove external fluoride from leaf surfaces (Franzaring et al., 2007). After drying in an oven at 65 °C for 5 days, tissues were analyzed for fluoride content. A comparison of fluoride tissue concentrations between control plants, which contained no detectable fluoride (< 8.5 µg F/g tissue dry weight), and the exposed tall fescue plant (870 µg F/g) confirmed enhanced plant fluoride uptake from HF dosing through the gas exposure system (Fig. 7).

Plant fluoride uptake varies based on factors such as plant species, plant age, fluoride dose, and duration of exposure. To evaluate if the enhanced fluoride uptake of tall fescue achieved during the 48-h HF exposure was within the range observed in bioindicator field applications, a general comparison was made to the reported fluoride content in grasses from multiple studies. Sant’Anna-Santos et al. (2021) reported fluoride concentrations in *Panicum maximum*, a tropical perennial grass, placed near an HF-emitting source to range from approximately 5 µg F/g to 14 µg F/g. Grasses were exposed to HF emissions that varied between 0.1 and 16 ppmv over 3 to 9 days. In studies using perennial ryegrass (*Lolium perenne)*, a closely related species to tall fescue, leaf fluoride concentrations of grass cultures in the vicinity of HF-emitting facilities ranged from 4.3 to 912 µg F/g (Rey-Asensio & Carballeira, 2007) and 14.5 to 700 µg F/g (Klumpp et al., 1994). In these two studies, perennial ryegrasses were exposed to HF concentrations of approximately 0.1 ppbv and 0.1 to 1 ppbv, respectively, over 28 days. Although there are differences in exposure conditions, this comparison demonstrates that (1) tall fescue exhibited visible stress symptoms at elevated fluoride tissue concentrations observed in field monitoring studies without significant injury confirming the results of Taylor et al. (1981) and (2) sufficient fluoride uptake occurred in tall fescue tissue using the gas exposure system despite system losses. Additional experiments will be conducted using the gas exposure system to examine the effects of chronic and acute HF exposures to tall fescue over a range of HF concentrations.

## Conclusion

A custom gas exposure system was constructed to support laboratory-based research on the use of plants as bioindicators for the detection and delineation of atmospheric HF releases. Despite significant HF losses to the exposure chamber, relatively stable HF concentrations in excess of 1 ppbv were achieved over 48 h. A preliminary 48-h plant exposure experiment confirmed that enhanced fluoride uptake occurred in tall fescue dosed with HF gas through the gas exposure system. Dosing concentrations, frequency, and duration of future experiments can be adapted using the gas exposure system to represent field conditions or emissions patterns. Additionally, the gas exposure system can be adapted in the future to research the effects of other relevant atmospheric pollutants on plants.

## Data Availability

Datasets generated during the system calibration experiments and the plant exposure experiment are available from the corresponding author upon reasonable request.
